# Urban-rural differences in transpiration patterns of *Pinus tabuliformis* and environmental drivers

**DOI:** 10.3389/fpls.2026.1861479

**Published:** 2026-05-29

**Authors:** Huanhuan Liu, Jia Wang, Xu Sun, Zhong Zheng, Weiqi Zhou

**Affiliations:** 1 School of Life Sciences, Division of Life Sciences and Medicine, University of Science and Technology of China, Hefei, China; 2 State Key Laboratory for Regional and Urban Ecology, Research Center for Eco-Environmental Sciences, Chinese Academy of Sciences, Beijing, China; 3 Beijing-Tianjin-Hebei Urban Megaregion National Observation and Research Station for Eco-Environmental Change, Research Center for Eco-Environmental Sciences, Chinese Academy of Sciences, Beijing, China; 4 University of Chinese Academy of Sciences, Beijing, China; 5 Xiongan Institute of Innovation, Xiongan New Area, China

**Keywords:** environmental regulation, *Pinus tabuliformis*, sap flow, transpiration, urban–rural difference

## Abstract

**Introduction:**

Tree transpiration plays a critical role in water cycling and energy balance, contributing to the regulation of urban microclimates. Rapid urbanization has created pronounced urban–rural environmental differences that can substantially alter tree transpiration and its regulatory mechanisms, yet these effects remain poorly understood.

**Methods:**

We investigated the transpiration patterns of *Pinus tabuliformis*, a native tree species in Beijing, based on a 10-year dataset (2014-2023) of sap flow and environmental measurements collected from contrasting urban and rural sites. Linear models, piecewise structural equation modeling (piecewise SEM), and boundary line analysis were used to quantify transpiration responses to environmental drivers across contrasting urban and rural conditions, identify environmental thresholds, and assess stomatal regulation strategies under contrasting environmental conditions.

**Results:**

The results showed that transpiration rates were significantly higher at the urban site, with mean daily rates approximately threefold greater than those at the rural site, and peak transpiration occurring 1.3 h earlier. The responses of transpiration to environmental drivers differed between sites. For example, transpiration increased with vapor pressure deficit (VPD) and plateaued at approximately 1.76 kPa at the urban site, whereas it increased initially but declined beyond a threshold of 1.33 kPa at the rural site. Transpiration at the urban site was primarily driven by photosynthetically active radiation (PAR), indicating energy-limited conditions, whereas at the rural site it was mainly constrained by soil moisture, reflecting water-limited conditions. These differences were associated with contrasting stomatal regulation strategies, with rural trees exhibiting higher sensitivity to VPD (m/g_cref_ = 0.86) and stronger stomatal control under water-limited conditions.

**Discussion:**

Our results demonstrate that long-term urban-rural environmental differences reshape transpiration patterns, environmental responses, and stomatal regulation strategies in trees. These findings provide new insights into tree water-use strategies under urbanization and suggest that atmospheric drought and soil water availability jointly regulate transpiration responses across contrasting habitats. Our study also provides key parameters for improving ecohydrological models and urban forest management.

## 1 Introduction

Transpiration is the process by which water is absorbed by roots, transported through xylem, and released into the atmosphere via leaf stomata (Sperry et al., 2002; Tyree and Zimmermann, 2002). As a key part of the soil-plant-atmosphere continuum (SPAC), transpiration regulates the hydrological cycle and land surface energy balance (Schlesinger and Jasechko, 2014; Maltese et al., 2018). Trees transfer water to the atmosphere and influence precipitation, temperature, cloud formation, and groundwater dynamics (Ellison et al., 2017). These changes affect the local and regional climate. In urban environments, transpiration also contributes to microclimate regulation and mitigation of the urban heat island effect (Manoli et al., 2019). Understanding the dynamics of tree transpiration and their responses to environmental factors is crucial for evaluating the effects of climate change on plant physiology and ecology.

Tree transpiration is influenced by multiple hydrometeorological factors, including air temperature, radiation, vapor pressure deficit (VPD), precipitation, and soil moisture (He et al., 2021; Ahongshangbam et al., 2023; Huang et al., 2024). With increasing radiation and temperature, transpiration rates generally increase (Patankar et al., 2015; Blasini et al., 2022). The response of transpiration to VPD exhibits a clear nonlinear pattern, whereby transpiration increases with rising VPD but is suppressed by stomatal closure once a threshold is exceeded (Song et al., 2022; Khadke et al., 2026). Soil moisture promotes transpiration by regulating hydraulic processes within the soil–plant–atmosphere continuum (SPAC) (Grossiord et al., 2020). Despite extensive research, the dominant drivers of transpiration remain debated, largely due to environmental heterogeneity across regions (Wang et al., 2024; Zhao and Fan, 2024; Anys and Weiler, 2025). Evidence indicates that both transpiration responses to environmental factors and stomatal regulation strategies vary across environments (Winbourne et al., 2020; Chen et al., 2023a, 2024b). For example, compared with water-limited regions, transpiration in humid regions shows stronger correlations with radiation and VPD (Zhao and Fan, 2024). Under sufficient soil moisture conditions, high VPD can significantly enhance transpiration (Rötzer et al., 2019). In contrast, under soil moisture deficit conditions, increasing VPD induces stomatal closure, reduces canopy conductance, and thereby suppresses transpiration (Denham et al., 2021; Song et al., 2022).

With ongoing climate change and urbanization, urban environments have become natural laboratories for simulating future climate conditions (Diamond and Martin, 2021). Urban and rural environments differ markedly in both thermal and hydrological conditions. For example, urban areas typically exhibit higher CO_2_ concentrations, greater resource availability, elevated temperatures, longer growing seasons, and increased radiation, all of which can promote tree growth (Winbourne et al., 2020; Liu et al., 2021). These environmental differences may reshape transpiration regulation mechanisms in urban settings compared to those in rural areas (McCarthy and Pataki, 2010). For example, elevated urban temperatures increase VPD, enhancing atmospheric water demand and stimulating transpiration (Novick et al., 2024). Changes in precipitation patterns and soil moisture deficits can constrain root water uptake, thereby suppressing transpiration (Grossiord et al., 2020; Wang et al., 2024). Understanding differences in transpiration and environmental responses between urban and rural trees is crucial for improving urban climate resilience and ecosystem management. Most previous studies, however, have focused on biophysical regulation of transpiration under natural conditions. Urban transpiration studies have mainly focused on short term observations of individual tree species (Shashua-Bar et al., 2023; Chen et al., 2024b) or comparisons among different species groups (Pataki et al., 2011; Stratópoulos et al., 2018; Anys and Weiler, 2025). However, the mechanisms linking tree transpiration to environmental drivers over the long term, especially within the same species across urban and rural settings, remain unclear (Hayat et al., 2022). In addition, there is still limited understanding of how urban-rural environmental differences influence transpiration regulation within the same species (Winbourne et al., 2020). It remains unclear how long-term environmental differences between urban and rural areas affect transpiration rate patterns, how these patterns are modulated by environmental drivers, and how stomatal regulation strategies may shift under contrasting conditions.

The contrast between irrigated urban habitats in Beijing and relatively water-limited rural forests represents a common environmental gradient across many rapidly urbanizing regions. Understanding how urban water management and atmospheric drought jointly regulate tree transpiration under contrasting environments may therefore provide broader insights into tree water-use strategies and urban ecosystem management under climate change. To better understand these mechanisms, we conducted a 10-year (2014–2023) monitoring study of *Pinus tabuliformis* in Beijing to investigate differences in transpiration rate patterns and their environmental controls across urban and rural environments. Specifically, we aim to examine: (1) whether transpiration rate patterns differ between urban and rural environments; and (2) whether the responses of transpiration to environmental factors differ under contrasting environmental conditions, and whether the dominant drivers of transpiration vary between environments.

## 2 Materials and methods

### 2.1 Study area and sample trees

The study was conducted at two contrasting sites in Beijing representing urban and rural habitats, namely the Beijing Teaching Botanical Garden (ZWY) and Mangshan National Forest Park (MS). The Beijing Teaching Botanical Garden (40°00′06.66″ N, 116°12′34.49″ E) is situated in the urban core of Beijing and serves as an educational base for primary and secondary school students. Covering approximately 11.65 hectares, the garden harbors more than 1,500 plant species, including a specialized tree taxonomy zone. Vegetation in this area is under intensive human management. In contrast, Mangshan National Forest Park (40°11′31.20″ N, 116°17′36.37″ E) is located in the northern rural area of Beijing, within Changping District, and in the Yanshan Mountain range. The site experiences relatively low human disturbance, with vegetation largely maintained in a natural or semi-natural state, making it representative of rural environmental conditions. The park reaches a maximum elevation of 659 meters. It covers about 13,000 hectares of plantation forest, with a forest coverage rate of 96.5%.

**Figure 1:**
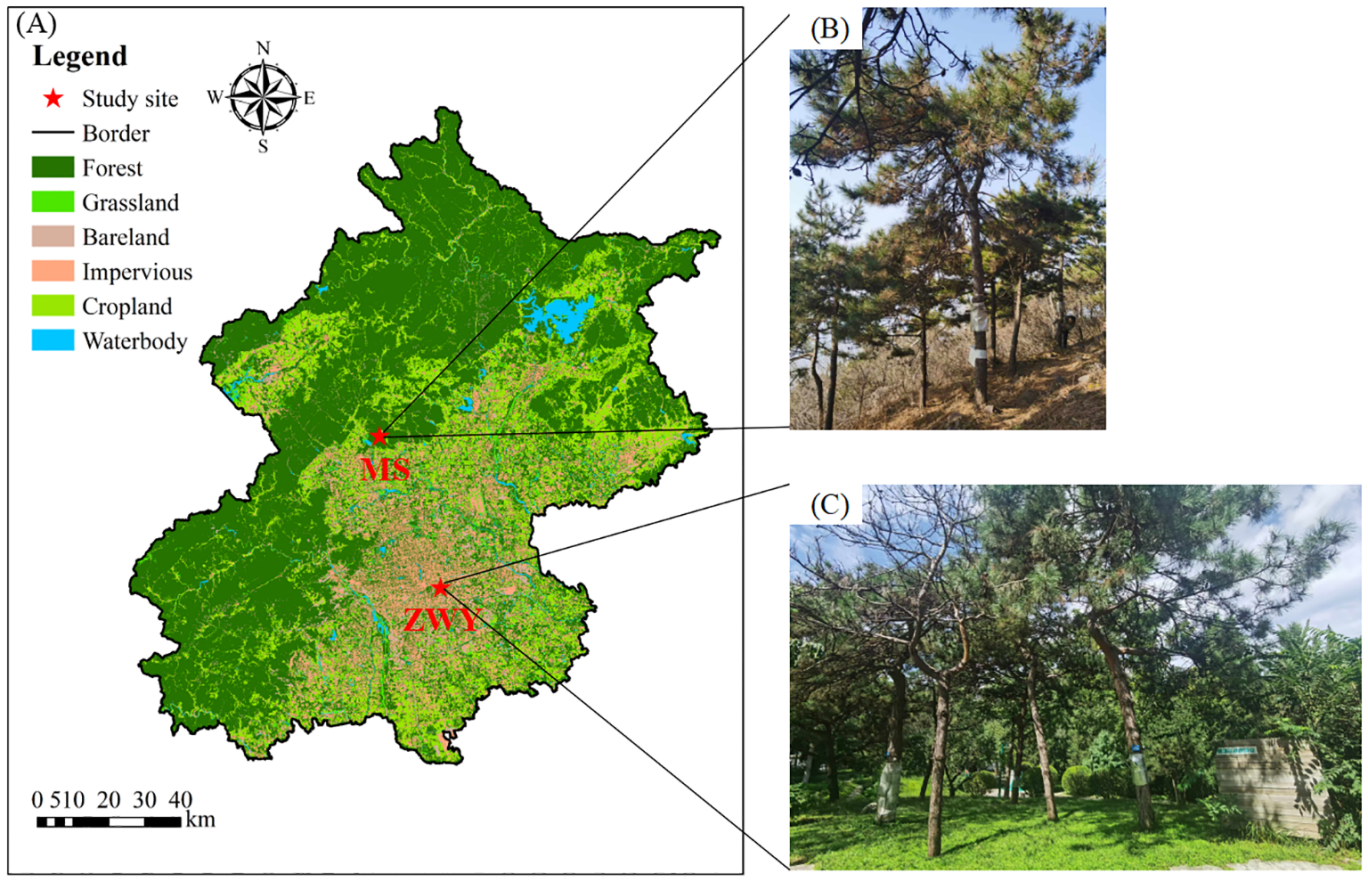
Location and tree samples in the study area. The Beijing Teaching Botanical Garden (ZWY) and Mangshan National Forest Park (MS) represent the urban and rural sites, respectively. **(A)** Spatial distribution of the urban and rural study sites in Beijing, China. **(B)** Sampled *Pinus tabuliformis* at the rural site. **(C)** Sampled *Pinus tabuliformis* at the urban site.

*Pinus tabuliformis*, a dominant native tree species in Beijing, was selected due to its widespread distribution across urban and rural environments and its adaptability to local conditions. This evergreen conifer, endemic to China, is widely distributed in water-limited regions of northern China (Song et al., 2023). It is light-demanding and deep-rooted, adapted to dry and cold climates, and tolerant of environmental stress, typically growing in deep, well-drained soils. For the urban - rural comparison, three healthy individuals were selected from the Beijing Teaching Botanical Garden (urban site) and three from the central plantation of Mangshan National Forest Park (rural site), representing typical individuals in each environment (**Figure 1**). To investigate differences in transpiration regulation and environmental responses of *Pinus tabuliformis* under long-term urban and rural conditions, sample trees were selected primarily based on study objectives, diameter at breast height (DBH), and overall growth. The mean age of urban trees was approximately 30 years, compared to approximately 50 years for rural trees. Urban sites experience greater vegetation turnover and anthropogenic management, resulting in younger urban trees, whereas trees at the rural site grow under more natural conditions and therefore tend to represent relatively older stands. According to forestry classification standards, Pinus tabuliformis stands are classified as middle-aged at 31–50 years under natural conditions and 21–30 years under intensive management (State Forestry Administration, 2018). Thus, despite the age difference between sites, both urban and rural trees in this study typically represent vigorous middle-aged stands with active physiological functioning (Guo et al., 2011).

### 2.2 Sap flow measurement and calculation

Sap flow was measured using thermal dissipation probes (Dynamax Inc., Houston, TX, USA). At each site, three healthy trees of similar size were selected for monitoring. Each sap flow sensor consisted of a pair of probes and a heating element. The probes were inserted into the sapwood with a vertical spacing of 10–15 cm, with the heated probe positioned above and the reference probe below. Sap flow was determined from the temperature difference between the two probes. For each sample tree, sensors (30 mm in length) were installed at breast height (1.3 m) on both the north- and south-facing sides of the stem. Measurements were recorded at 10-min intervals, and mean values were stored in a data logger and transmitted in real time via the General Packet Radio Service (GPRS) system.

Quality control of sap flow data was performed according to the following criteria:(1) the temperature difference (ΔT) ranged between 4 and 15 °C, with a daily amplitude< 5 °C;(2) abnormal peak values were removed;(3) data with an effective measurement duration< 4 h were excluded. Zero-flow conditions were identified based on nighttime periods (20:00-05:50) when VPD remained below 0.1 kPa for at least 2 h (Oishi et al., 2016; Ward et al., 2017; Speckman et al., 2020).

Sap flow density (F_d_, g cm^-^² s^-^¹) was calculated as:


$${\text{F}}_{\text{d}}=0.0119\times {\text{K}}^{1.231}$$

$$K=\frac{\Delta T_{0}-\Delta T}{\Delta T}$$

where K is a dimensionless empirical coefficient, ΔT (°C) is the temperature difference between the heated and reference probes, and ΔT_0_ (°C) is the maximum temperature difference under zero-flow conditions.

The whole-tree transpiration rate (Tr, g s^-^¹) was calculated by scaling sap flow density with sapwood area (As, cm²):


$$T_{r}=F_{d}\times A_{s}$$

Daytime canopy stomatal conductance per unit sapwood area (G_As_, mol m^-^² s^-^¹) was estimated as (Flo et al., 2022):


$$G_{A_{s}}=\frac{\left(115.8+0.4326T_{a}\right)F_{d}}{VPD}\times \eta \times \frac{T_{0}}{T_{0}+T_{a}}e^{-0.00012h}$$

where Ta is air temperature (°C), VPD is vapor pressure deficit (kPa), *η* is a constant (44.6 mol m^-^³), T_0_ is the reference temperature (273 K), and h is site elevation(m).

### 2.3 Tree sapwood area measurement

**Figure 2:**
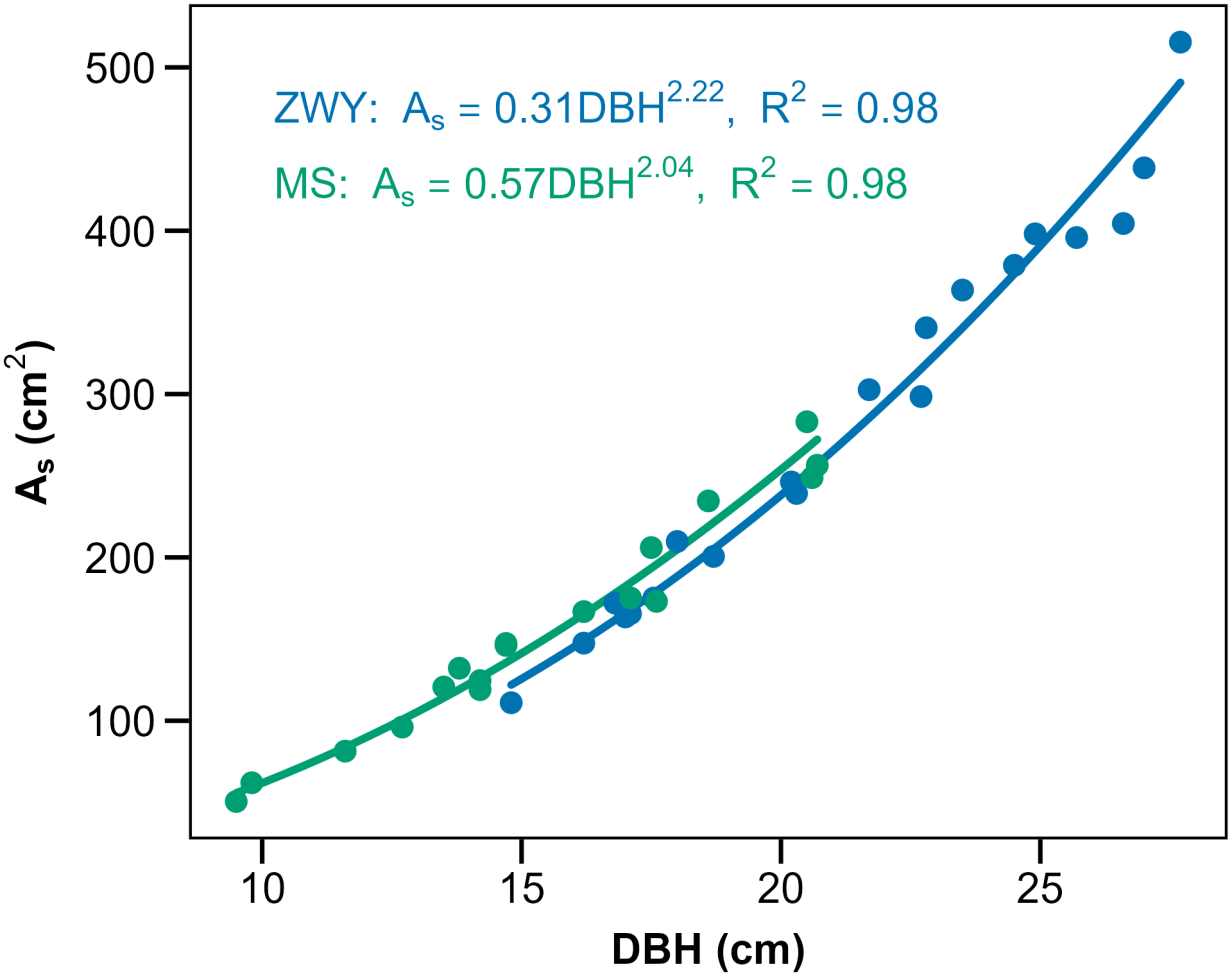
The relationship between sapwood area (As) and diameter at breast height (DBH) for *Pinus tabuliformis*.

To avoid damaging sample trees, we collected increment cores from the same species growing nearby under similar conditions. Sapwood area was determined with a staining method (Spicer and Gartner, 2001). We established allometric relationships between diameter at breast height and sapwood area using power functions. Separate equations were developed for urban and rural *Pinus tabuliformis* (**Figure 2**). We estimated annual sapwood area by combining yearly DBH with allometric equations. **Table 1** presents tree structural parameters for *Pinus tabuliformis* at urban (ZWY) and rural (MS) sites from 2014 to 2023.

**Table 1:** Structural parameters of urban and rural *Pinus tabuliformis*.

Year	ZWY		MS	
	DBH	As	DBH	As
2014	21.94 ± 1.27	295.02 ± 37.70	16.16 ± 0.38	164.66 ± 7.77
2015	22.48 ± 1.24	311.13 ± 38.50	16.36 ± 0.30	168.65 ± 6.22
2016	22.86 ± 1.25	322.92 ± 39.62	16.59 ± 0.21	173.66 ± 4.54
2017	23.25 ± 1.23	335.22 ± 39.91	16.82 ± 0.17	178.44 ± 3.70
2018	23.65 ± 1.19	348.15 ± 39.61	16.97 ± 0.15	181.80 ± 3.29
2019	24.06 ± 1.18	361.30 ± 40.30	17.10 ± 0.15	184.60 ± 3.24
2020	24.41 ± 1.17	373.03 ± 40.74	17.22 ± 0.12	187.10 ± 2.60
2021	24.76 ± 1.12	384.90 ± 39.57	17.36 ± 0.09	190.30 ± 2.11
2022	25.37 ± 1.13	405.97 ± 41.09	17.71 ± 0.16	198.15 ± 3.67
2023	25.93 ± 1.02	425.87 ± 37.60	18.21 ± 0.20	204.00 ± 3.72

The diameter at breast height (DBH, cm) and sapwood area (As, cm^2^) (mean value ± standard error) of *Pinus tabuliformis* at both urban and rural sites.

### 2.4 Environmental factors measurement

To characterize environmental conditions at both sites, we installed automatic weather stations and soil moisture sensors at the Beijing Teaching Botanical Garden (ZWY) and Mangshan National Forest Park (MS). Weather stations were placed near sampled trees and continuously recorded air temperature (HMP45C, Vaisala Inc., Finland), relative humidity (HMP45C, Vaisala Inc., Finland), wind speed (034B, Met One Instruments, USA), photosynthetically active radiation (LI190R, LI-COR, USA), and precipitation (TE525MM, Campbell Scientific, USA). Meteorological variables were synchronously monitored at both sites every 10 minutes. Air temperature, relative humidity, wind speed, photosynthetically active radiation(PAR, μ mol/m^2^/s), and precipitation were then used for subsequent environmental response analyses. To track soil water dynamics near sampled trees, we installed soil temperature and moisture sensors at both sites. We measured soil moisture at 10–50 cm depth and recorded data hourly using a data logger. Vapor pressure deficit (VPD, kPa) was calculated from Ta and RH (Campbell and Norman, 1998):


$$VPD=0.611\times \frac{17.27\times T_{a}}{T_{a}+237.3}\left(1-RH\right)$$

**Figure 3:**
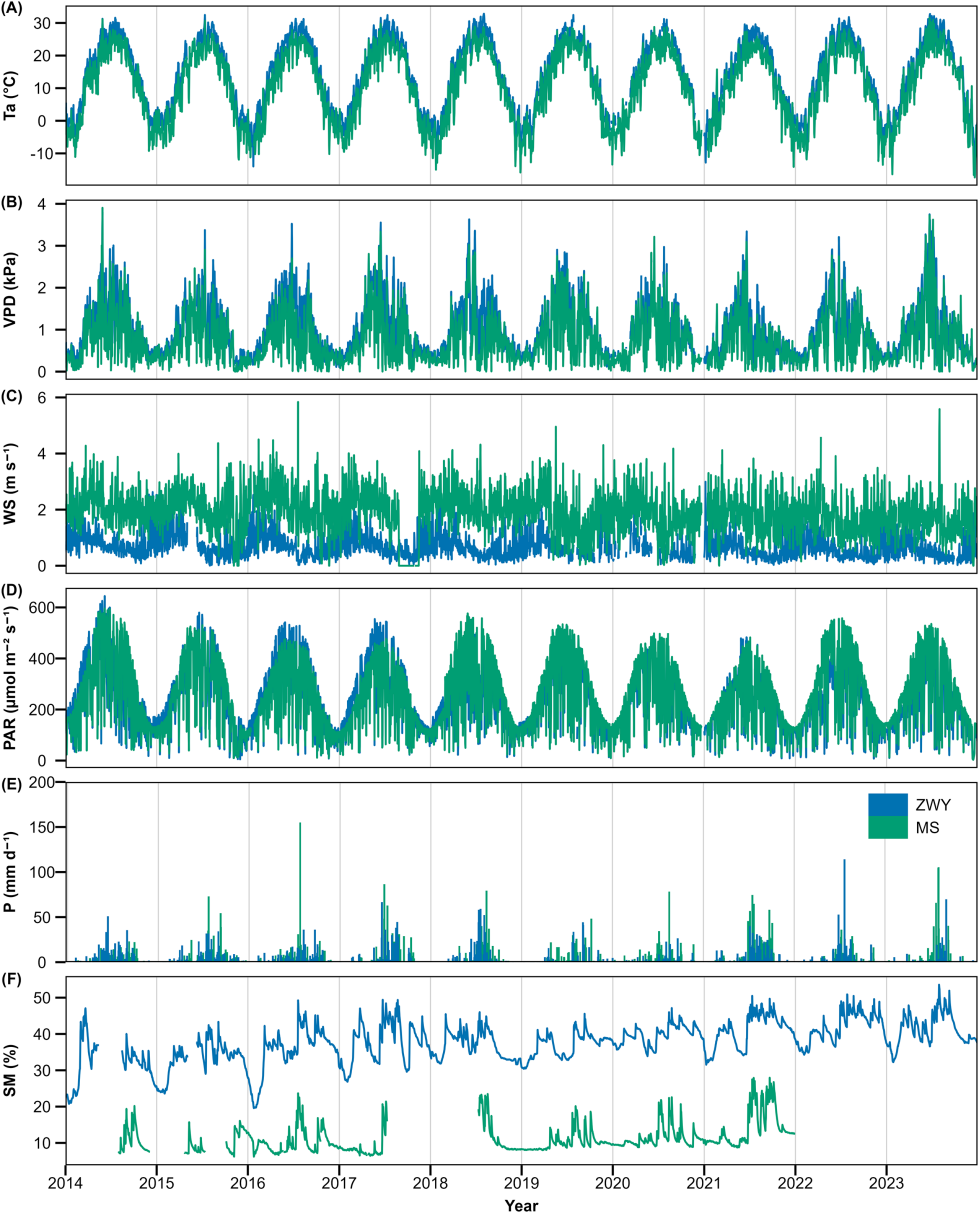
Daily variations of environmental factors from 2014 to 2023. Ta **(A)**, VPD **(B)**, WS **(C)**, PAR **(D)**, P **(E)**, and SM **(F)** represent air temperature, vapor pressure deficit, wind speed, photosynthetically active radiation, precipitation, and soil moisture, respectively.

The urban site, with higher air temperature and VPD but lower wind speed, experienced stronger atmospheric drought stress and weaker ventilation than the rural site. Although it received less precipitation, the urban site still had significantly higher soil moisture due to regular irrigation (**Figure 3**). This highlights the crucial role of anthropogenic management in regulating soil water availability in urban ecosystems.

### 2.5 Statistical analysis

Paired Wilcoxon signed-rank tests were applied to assess differences in environmental variables and transpiration rates between urban and rural sites. To characterize the response patterns and strength of transpiration to environmental drivers, linear models, Gompertz growth models, and generalized additive models (GAM) were fitted. The optimal model for each response was selected based on the Akaike Information Criterion corrected (AICc) for small sample sizes. For nonlinear responses, response thresholds were quantified using 95% of the asymptotic value derived from the Gompertz model and the first derivative of the GAM smoothing function. These thresholds were used to identify sensitive and saturation ranges of transpiration under varying environmental conditions.

To disentangle the direct and indirect effects of environmental variables on transpiration, we constructed piecewise structural equation models (piecewise SEM) following (Lefcheck, 2016). The SEM model framework was developed based on the SPAC theory and previous related studies (Fan et al., 2016; Chen et al., 2023a; Fang et al., 2025). We hypothesized that environmental factors influence transpiration rate (Tr) both directly and indirectly through interactions among variables. Specifically, we hypothesized that PAR regulates Tr both directly and indirectly through its effects on Ta, VPD and soil moisture. Wind speed was assumed to influence Ta and VPD, while Ta further regulates VPD and soil moisture. VPD was assumed to directly affect transpiration through atmospheric water demand and indirectly regulate Tr through its influence on soil moisture. Soil moisture was assumed to directly regulate Tr by constraining plant water supply. Based on these hypothesized relationships, transpiration rate was specified as the response variable, and PAR, Ta, VPD, soil moisture, and wind speed were included to establish the SEM pathways. To account for interannual variability, the year was included as a random effect in all component models. The models were used to quantify the direct, indirect, and total effects of environmental drivers.

To assess the sensitivity of canopy stomatal conductance (G_As_) to VPD, we used boundary line analysis (Chen et al., 2023a). Under well-coupled conditions, Gs shows an approximately linear relationship with ln(VPD) (Oren et al., 1999):


$$G_{A_{s}}=-m\ln\left(VPD\right)+g_{cref}$$

where m is the regression slope representing the sensitivity of G_As_ to VPD, with larger values indicating faster stomatal closure; g*_cref_* is the reference canopy conductance at VPD = 1 kPa, reflecting potential transpiration capacity; and the ratio m/g*_cref_* represents the relative sensitivity of Gs to atmospheric drought. A value of 0.6 indicates isohydric regulation, whereas higher values indicate greater stomatal sensitivity.

To ensure data quality, periods with continuous missing data exceeding three days were excluded. All analyses of transpiration responses were conducted at the daily scale during the growing season (May-October) to minimize the influence of leaf phenology. Data affected by rainfall (Rain > 0mm) were excluded to avoid interference from precipitation events (Bachofen et al., 2023). All data processing, visualization, and statistical analyses were performed using R (version 4.3.3) and RStudio (version 2024.12.1 + 563).

## 3 Results

### 3.1 Differences in transpiration rate between urban and rural sites

**Figure 4:**
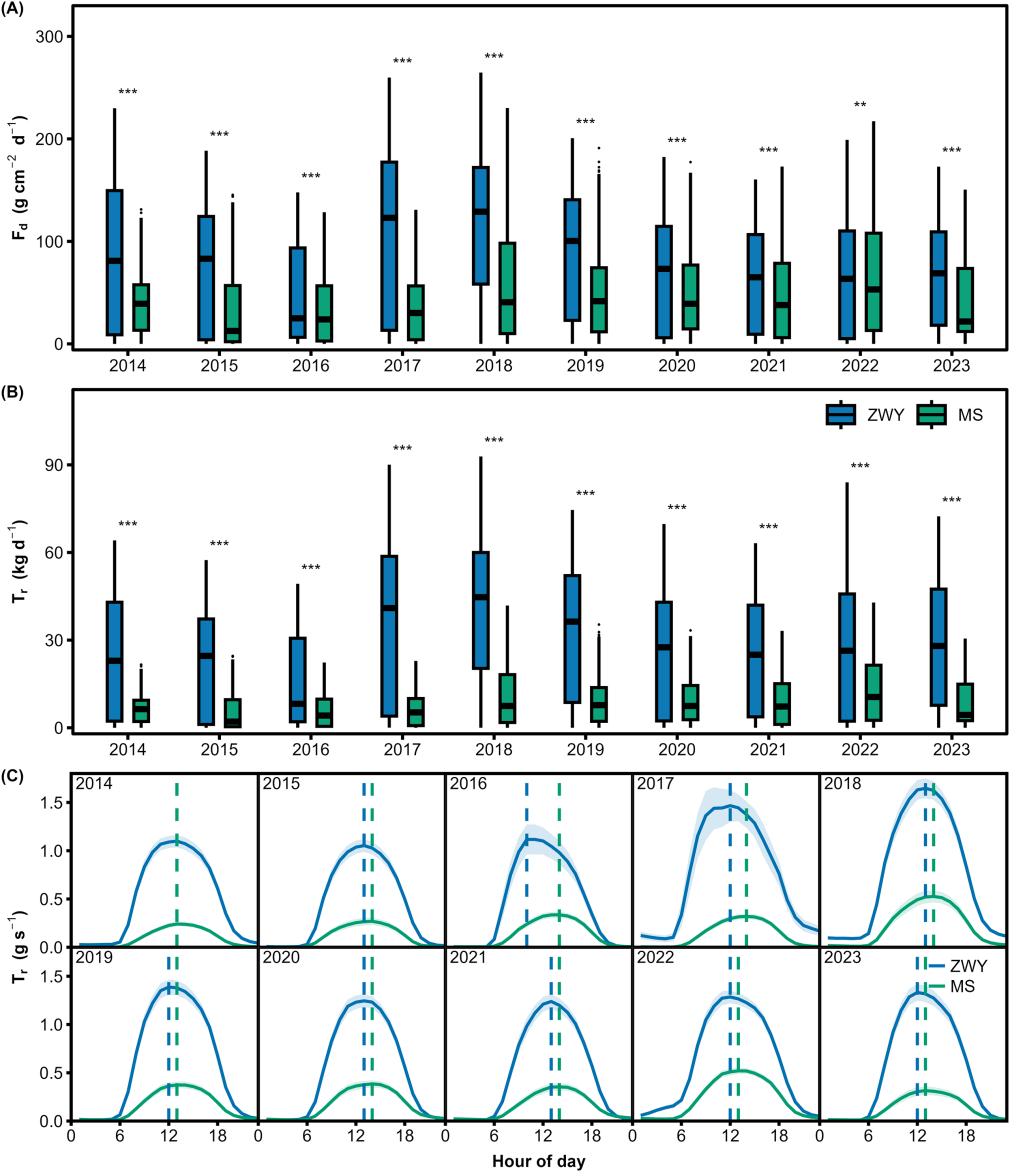
Interannual differences in sap flow density **(A)**, transpiration rate **(B)** and diurnal dynamics **(C)** between the urban and rural sites. In **(A, B)**, boxplots show median, interquartile range and 1.5× IQR. Asterisks indicate significance of paired Wilcoxon tests between sites within each year (*P < 0.05, **P < 0.01, ***P < 0.001). In **(C)**, mean diurnal transpiration during the growing season (May–September) is shown. Lines show hourly means, shaded areas indicate 95% confidence intervals, and dashed lines mark peak timing.

Sap flow density and transpiration rate (Tr) of *Pinus tabuliformis* were significantly higher at the urban site than the rural site, and these differences remained stable from 2014 to 2023 (**Figure 4**). The mean difference in sap flow density between sites was 33.12 g cm^-^² day^-^¹, with higher values at the urban site. Mean daily Tr per tree was approximately 29.1 kg day^-^¹ at the urban site and 8.8 kg day^-^¹ at the rural site, indicating that transpiration at the urban site was about three times higher than at the rural site. The difference in Tr exceeded that in sap flow density.

At the diurnal scale during the growing season, transpiration rates at both sites showed a clear unimodal pattern, but the timing of peak Tr differed (**Figure 4**). The peak Tr occurred around midday (10:00–14:00), with an earlier peak at the urban site by approximately 1.3 h. Overall, transpiration was consistently higher in urban trees.

### 3.2 Responses of transpiration to environmental factors

**Figure 5:**
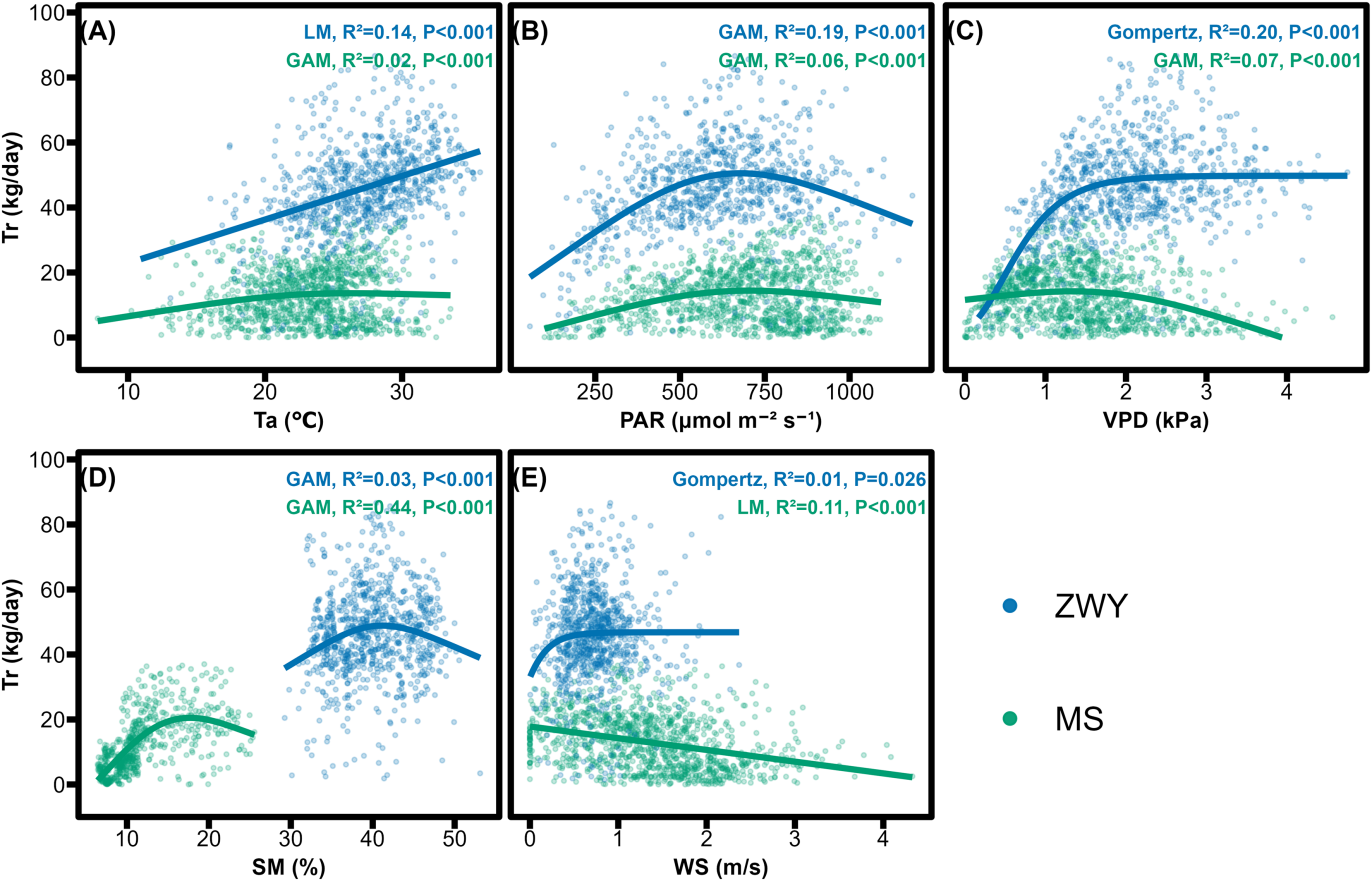
Relationships between transpiration rate and environmental variables in *Pinus tabuliformis* at urban and rural sites. Ta **(A)**, PAR **(B)**, VPD **(C)**, SM **(D)**, and WS **(E)** represent air temperature, photosynthetically active radiation, vapor pressure deficit, soil moisture, and wind speed, respectively. LM, Gompertz, and GAM represent linear models, Gompertz growth models, and generalized additive models, respectively.

The responses of transpiration rate in *Pinus tabuliformis* to environmental variables differed between urban and rural sites in both patterns and threshold values (**Figure 5**). At the urban site, Tr showed a linear relationship with air temperature, while at the rural site, Tr exhibited a unimodal response. At the urban site, Tr increased with VPD and then saturated; at the rural site, Tr first increased and then declined as VPD rose. At the rural site, the response of Tr to Ta peaked at 27.7 °C. The VPD threshold for Tr was higher at the urban site, reaching saturation near 1.76 kPa; at the rural site, the inflection point occurred at 1.33 kPa. Tr at the urban site showed weak responses to wind speed. At the rural site, Tr decreased linearly with increasing wind speed. Meanwhile, GAM analysis showed that transpiration rate at both sites exhibited unimodal responses to PAR and soil moisture. The PAR response inflection point was about 676 μmol m^-^² s^-^¹ at the urban site and 707 μmol m^-^² s^-^¹ at the rural site. Tr at the urban site was weakly affected by soil moisture, whereas at the rural site it showed a threshold response, with an inflection point at about 17.7%.

### 3.3 Pathways of environmental regulation on transpiration

**Figure 6:**
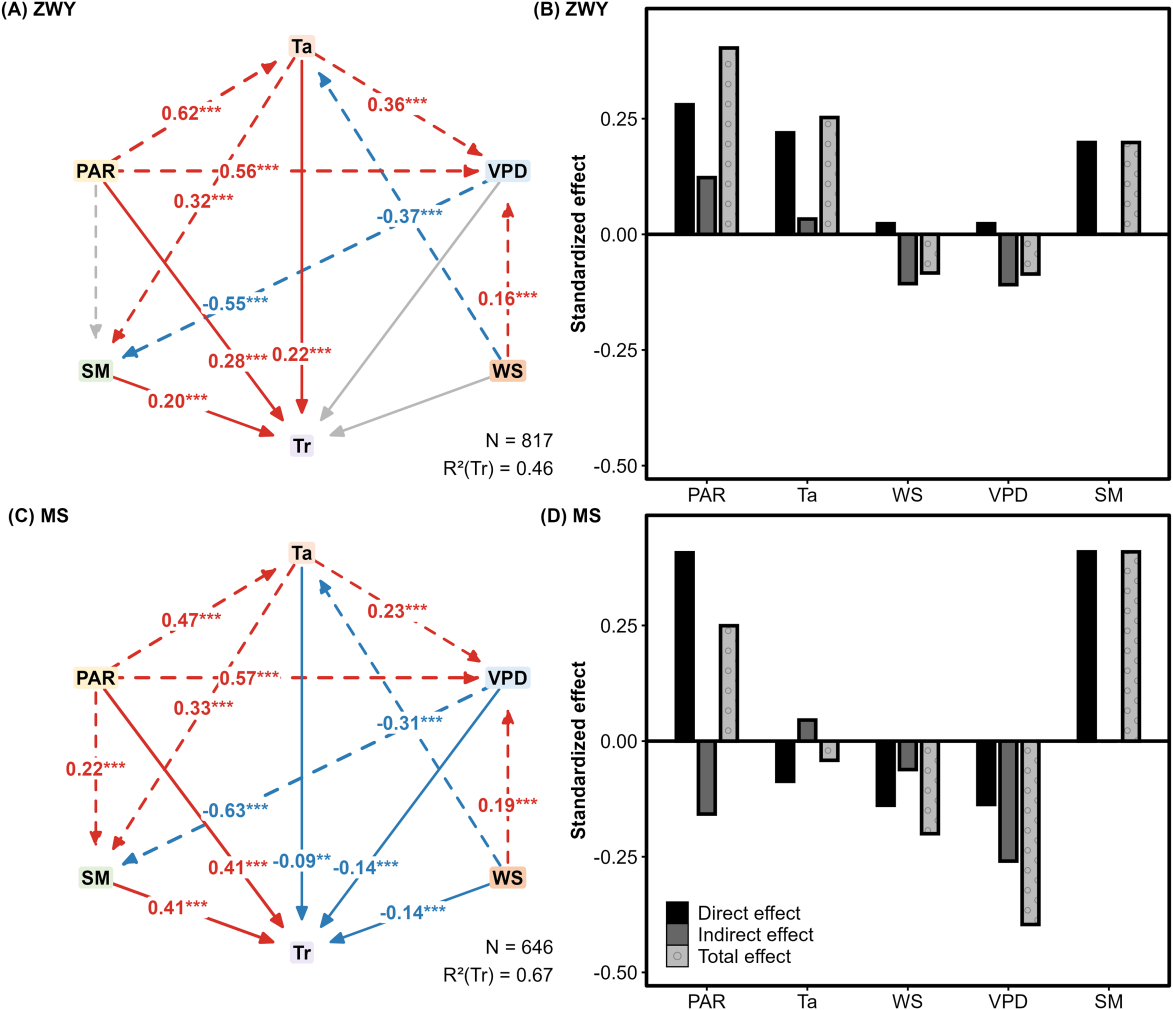
Direct and indirect effects of environmental factors on *Pinus tabuliformis* transpiration rate (Tr) at urban and rural sites. Piecewise structural equation models for the urban site **(A)** and rural site **(C)**, and standardized effect of environmental factors on Tr at the urban site **(B)** and rural site **(D)**. Solid arrows indicate direct pathways, whereas dashed arrows indicate indirect pathways. Red and blue arrows represent positive and negative effects, respectively. Numbers beside the arrows are standardized path coefficients, and the asterisks attached to the coefficients indicate significance levels (*P < 0.05, *P* < 0.01, *P < 0.001). N represents the sample size used for the piecewise structural equation models. ZWY: Fisher’s C = 20.16, df = 2, *P* < 0.05, AIC = 46.2;MS: Fisher’s C = 11.72, df = 2, *P* < 0.05, AIC = 37.7.

The regulatory pathways of environmental variables on transpiration rate differed markedly between the urban and rural sites (**Figure 6**). At the urban site, PAR was the dominant driver of Tr (β = 0.40), accounting for the largest total contribution (39.37%), while at the rural site, soil moisture emerged as the main controlling factor (β = 0.41), contributing 31.55% of the total effect.

Regarding direct influences, PAR exerted the strongest positive effect on Tr at the urban site, contributing 20.01%. Meanwhile, at the rural site, soil moisture had the largest direct positive effect on Tr, accounting for 17.81%. In addition, VPD and wind speed had a direct negative effect on Tr at the rural site, but this effect was not significant at the urban site. For indirect effects, the most important pathway at the urban site involved PAR indirectly enhancing Tr by increasing air temperature, accounting for 9.67% of Tr. By contrast, at the rural site, VPD indirectly reduced Tr by suppressing soil moisture (11.29%). Overall, the environmental variables included in the structural equation models explained 46% of the variation in Tr at the urban site and 67% at the rural site.

## 4 Discussion

### 4.1 Urban-rural divergence in transpiration patterns and environmental regulation

We found that transpiration rates were significantly higher at the urban site than at the rural site. This result is consistent with previous studies reporting enhanced transpiration in urban environments (Asawa et al., 2017). For instance, Ouyang found that urban trees exhibit higher transpiration rates than their rural counterparts, primarily due to enhanced hydraulic capacity and nutrient availability associated with urbanization (Ouyang et al., 2022). In contrast, Thomsen reported lower transpiration rates in urban trees than in suburban trees, attributing this to higher VPD, reduced photosynthetically active radiation, and lower soil moisture at the urban site (Thomsen et al., 2020). Under these conditions, elevated VPD induces stomatal closure, thereby suppressing transpiration. In our study, we observed that the urban site exhibited higher air temperatures and soil moisture, both of which promoted transpiration. Additionally, the sapwood area of *Pinus tabuliformis* was greater at the urban site than at the rural site, further amplifying the difference in transpiration rate. Previous studies have also demonstrated that improved water and nutrient availability in urban environments can enhance tree growth, resulting in larger sapwood area and greater water transport capacity (Moser et al., 2017; Liu et al., 2021).

Furthermore, we found that PAR dominates transpiration at the urban site, while soil moisture drives transpiration at the rural site. This pattern indicates a shift from energy-limited transpiration at the urban site to water-limited transpiration at the rural site. This result aligns with prior studies identifying radiation as the primary driver of transpiration in urban trees (Clausnitzer et al., 2011; Anys and Weiler, 2025). In contrast, rural trees experience lower soil moisture and prolonged water limitation, which likely alters the dominant controls on transpiration. Similarly, previous reports from water-limited systems indicate that soil moisture is the main limiting factor (Li et al., 2017; Fang et al., 2019; Kannenberg et al., 2024).

The transpiration response to environmental factors also differs between sites. As VPD increases, transpiration initially rises; once VPD exceeds a threshold, stomata close, reducing transpiration and preventing excessive declines in water potential that could cause xylem embolism (Ghimire et al., 2018; Grossiord et al., 2020). We found that this threshold is higher at the urban site, suggesting urban trees sustain transpiration at higher VPD, likely due to greater soil water availability. This aligns with earlier findings showing stomatal closure at lower VPD under water stress, but shifting to higher VPD when soil water is ample (Chen et al., 2023a). We also found that transpiration at the rural site is limited by wind speed. Wind affects transpiration indirectly by cooling leaves and altering VPD (Kim et al., 2014; Augustaitis and Pivoras, 2024). Over time, persistent high winds may potentially increase hydraulic tension and risk. This can promote a more conservative hydraulic structure and stomatal behavior, thereby limiting transpiration and reducing the risk of hydraulic failure (He et al., 2025).

### 4.2 Stomatal regulation under contrasting environmental conditions

**Figure 7:**
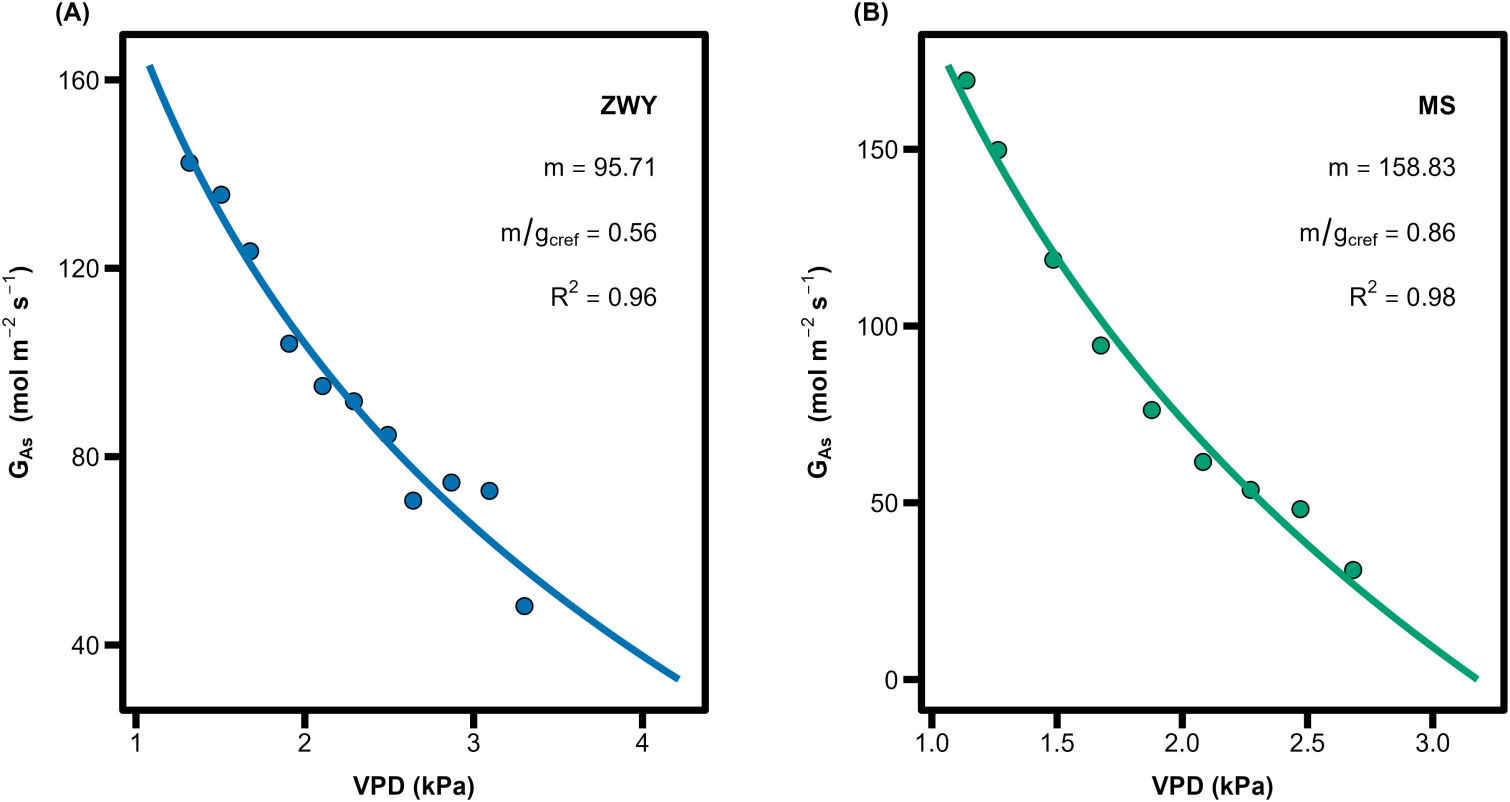
Responses of G_As_ to VPD in *Pinus tabuliformis* at urban **(A)** and rural **(B)** sites.

Stomatal sensitivity played a central role in mediating the observed differences in transpiration between sites. At both sites, canopy conductance declines nonlinearly with increasing VPD (**Figure 7**), consistent with the Oren framework (Oren et al., 1999). Compared with the urban site, *Pinus tabuliformis* at the rural site exhibited higher sensitivity(m) of G_As_ to VPD and lower reference canopy conductance (g_cref_). At the urban site, the relative sensitivity (m/g_cref_) was close to 0.6. In contrast, the value at the rural site (m/g_cref_=0.86) was substantially higher, indicating a stronger stomatal control. Similar patterns have been observed in water-limited regions, where *Pinus tabuliformis* exhibits stricter stomatal regulation (Song et al., 2023). Specifically, *Pinus tabuliformis* is generally considered a relatively isohydric species (Dang et al., 2021; Chen et al., 2024a; Chen and Wei, 2025). It regulates transpiration through tight stomatal control, thereby maintaining stable leaf water potential and avoiding hydraulic failure. During periods of soil drought, strict stomatal regulation helps maintain stable water potential gradients and hydraulic safety in conifers (Chen et al., 2023b, 2023a). In our study, rural trees are more strongly limited by soil moisture than urban trees. Long-term drought conditions appear to promote a conservative water use strategy. By tightly regulating stomatal opening, trees reduce transpiration rates and avoid excessive dehydration that could lead to xylem embolism.

### 4.3 Implications, limitations, and future perspectives

Tree age and developmental stage may also partially influence transpiration characteristics, hydraulic architecture, and stomatal regulation strategies. Previous studies have shown that younger trees often exhibit higher transpiration rates and water use. This is due to higher leaf area index (LAI) and greater stomatal conductance (Delzon et al., 2004; Delzon and Loustau, 2005; Buckley et al., 2012). In addition, transpiration and stomatal conductance generally decline with increasing tree age (Oishi et al., 2020). Older trees tend to rely more on deep soil water, so they may experience greater water stress during drought (Huo et al., 2018). Therefore, ontogenetic effects cannot be completely excluded in urban-rural comparisons. However, according to standard forestry classifications, both the urban and rural trees in this study represented vigorous middle-aged stands with active physiological functioning. Moreover, despite being younger, urban trees consistently exhibited larger DBH and sapwood area than rural trees throughout the monitoring period, suggesting that long term urban environmental conditions and anthropogenic management substantially influenced tree growth, hydraulic structure, and water transport capacity. More importantly, the observed urban-rural differences were reflected not only in transpiration rates, but also in environmental response patterns, dominant controlling factors, and stomatal sensitivity to VPD, suggesting that long term environmental differences remained the major drivers of transpiration regulation strategies between sites. Therefore, future studies should incorporate tree samples of similar age and size to better understand the effects of tree age on transpiration and water-use strategies, and to provide a scientific basis for the management of urban trees at different developmental stages.

Our findings suggest that urbanization can reshape tree water-use strategies under climate change. The shift from energy-limited transpiration in urban environments to water-limited transpiration in rural environments highlights how urbanization alters the dominant controls on tree transpiration through changes in soil water availability and atmospheric drought. Such contrasts between managed urban habitats and water-limited rural forests are becoming increasingly common across rapidly urbanizing regions worldwide. These findings also have important implications for tree management. As global climate change intensifies, trees will increasingly face combined stresses of extreme heat and soil drought. Accelerated soil moisture depletion may further limit the growth and transpiration of rural trees. In contrast, urban trees may require increased irrigation to maintain transpiration and cooling functions under higher temperatures and greater atmospheric drought. Tree management should therefore account for local environmental heterogeneity and optimize irrigation strategies to balance water use and cooling benefits. Although this study focused on Beijing, the mechanisms identified here may also be relevant to cities experiencing simultaneous warming, increasing atmospheric drought, and altered soil water availability under urbanization. Nevertheless, urban-rural differences in transpiration are likely to vary among climate zones, species, and management regimes. For example, in arid regions, urban trees may experience both elevated temperatures and reduced soil moisture, potentially inhibiting transpiration. Therefore, future research should include a range of climate zones and tree species, and systematically analyze the regulatory mechanisms of tree transpiration to enhance the generalizability of research conclusions and provide a stronger scientific basis for urban vegetation management in the context of climate change.

## 5 Conclusions

This study demonstrates that long-term urban-rural environmental differences reshape transpiration dynamics and regulatory mechanisms in *Pinus tabuliformis*. Urban trees had much higher transpiration rates, with daily values about three times those at the rural site and an earlier peak. These differences were linked to environmental controls: urban transpiration was driven by PAR, showing energy-limited conditions, while rural transpiration was constrained by soil moisture, reflecting water-limited conditions. Transpiration responses to environmental drivers also diverged between sites: urban trees had higher VPD thresholds and sustained transpiration as atmospheric demand increased. In contrast, rural trees reduced transpiration beyond lower thresholds. These patterns stemmed from differences in stomatal regulation: rural trees had greater stomatal sensitivity to VPD (m/g_cref_ = 0.86), indicating stronger control under water-limited conditions. Overall, findings point to a shift in transpiration regulation along the urban–rural gradient, from energy-limited to water-limited regimes. For management, maintaining soil moisture is essential for urban tree transpiration, while alleviating soil water limitation helps rural tree resilience. Future studies should expand these analyses across species and climates to clarify how general these mechanisms are and to improve predictions of tree water use under climate change.

## Data Availability

The raw data supporting the conclusions of this article will be made available by the authors, without undue reservation.
